# Value of virtual non-contrast images to identify uncomplicated cystic renal lesions: photon-counting detector CT vs. dual-energy integrating detector CT

**DOI:** 10.1007/s11547-024-01801-2

**Published:** 2024-03-21

**Authors:** Stephan Rau, Alexander Rau, Thomas Stein, Muhammad Taha Hagar, Sebastian Faby, Fabian Bamberg, Jakob Weiss

**Affiliations:** 1 Department of Diagnostic and Interventional Radiology, Medical Center, Faculty of Medicine, University of Freiburg, Hugstetter Str. 55, 79106 Freiburg, Germany; 2 Siemens Healthcare GmbH, Siemensstr. 3, 91301 Forchheim, Germany

**Keywords:** Tomography, X-Ray Computed, Renal lesions, Virtual non-contrast, Photon-Counting CT

## Abstract

**Purpose:**

To investigate the value of photon-counting detector CT (PCD-CT) derived virtual non-contrast (VNC) reconstructions to identify renal cysts in comparison with conventional dual-energy integrating detector (DE EID) CT-derived VNC reconstructions.

**Material and methods:**

We prospectively enrolled consecutive patients with simple renal cysts (Bosniak classification—Version 2019, density ≤ 20 HU and/or enhancement ≤ 20 HU) who underwent multiphase (non-contrast, arterial, portal venous phase) PCD-CT and for whom non-contrast and portal venous phase DE EID-CT was available. Subsequently, VNC reconstructions were calculated for all contrast phases and density as well as contrast enhancement within the cysts were measured and compared. MRI and/or ultrasound served as reference standards for lesion classification.

**Results:**

19 patients (1 cyst per patient; age 69.5 ± 10.7 years; 17 [89.5%] male) were included. Density measurements on PCD-CT non-contrast and VNC reconstructions (arterial and portal venous phase) revealed no significant effect on HU values (*p* = 0.301). In contrast, a significant difference between non-contrast vs. VNC images was found for DE EID-CT (*p* = 0.02). For PCD-CT, enhancement for VNC reconstructions was < 20 HU for all evaluated cysts. DE EID-CT measurements revealed an enhancement of > 20 HU in five lesions (26.3%) using the VNC reconstructions, which was not seen with the non-contrast images.

**Conclusion:**

PCD-CT-derived VNC images allow for reliable and accurate characterization of simple cystic renal lesions similar to non-contrast scans whereas VNC images calculated from DE EID-CT resulted in substantial false characterization. Thus, PCD-CT-derived VNC images may substitute for non-contrast images and reduce radiation dose and follow-up imaging.

## Introduction

Cystic renal lesions are a common incidental finding in cross-sectional imaging, relevant lesions larger 1 cm in diameter can be detected in up to 12–14% of all studies [1–4]. Accurate and reliable characterization of such lesions is essential for appropriate clinical management, as it allows for differentiating benign simple fluid-filled cysts from potentially malignant lesions, such as renal cell carcinoma [5, 6]. Currently, the Bosniak classification is the most widely used and accepted approach for cystic renal lesion characterization based on distinct imaging features such as morphology (i.e., septa and calcifications), density and contrast enhancement [7]. Unlike the morphological criteria, which are biased by some degree of subjectivity, density measurement and contrast enhancement are objective features, which provide a quantitative assessment. To facilitate the most accurate measurements, guidelines recommend CT protocols for renal lesions characterization to include true non-contrast images as well as contrast-enhanced series in the corticomedullary and nephrogenic phase [6, 8]. However, as renal lesions are frequently encountered as incidental findings in examinations acquired for various indications, not all series necessary for the reliable characterization of renal lesions are available, which may lead to further imaging-workup [9].

In this context, dual-energy integrating detector CT (DE EID-CT) with the possibility to calculate virtual non-contrast (VNC) images based on the unique linear attenuation coefficient of each substance was thought to provide additional information about renal lesion density and contrast enhancement if true non-contrast images were not available [10, 11]. However, previous studies reported inconclusive findings regarding the accuracy of HU measurements using DE EID-CT-derived VNC images with the risk of misclassifying lesions by over- or underestimating their true density and contrast enhancement [12, 13]. Therefore, DE EID-CT VNC images were not able to substitute for true non-contrast images in the workup of renal lesions so far.

With the recent implementation of photon-counting detector CT (PCD-CT) into clinical routine, a novel technology for data acquisition and reconstruction has become available with the potential to fundamentally change current workflows. PCD-CT employs energy-resolving detectors that directly count the number of photons at different energy levels, and thus facilitate energy discrimination along with the potential to reduce radiation dose [14, 15]. In theory, this allows for calculating more accurate VNC images in comparison with DE EID-CT, which could overcome current limitations for renal lesion characterization if no true non-contrast images are available.

Therefore, the aim of this study was to investigate the clinical value of PCD-CT-derived VNC images for simple cystic renal lesion characterization compared to VNC images from conventional DE EID-CT. Our hypothesis was that PCD-CT allows for calculating more accurate VNC images facilitating a more reliable characterization compared to DE EID-CT.

## Material and methods

### Patient cohort

**Fig. 1:**
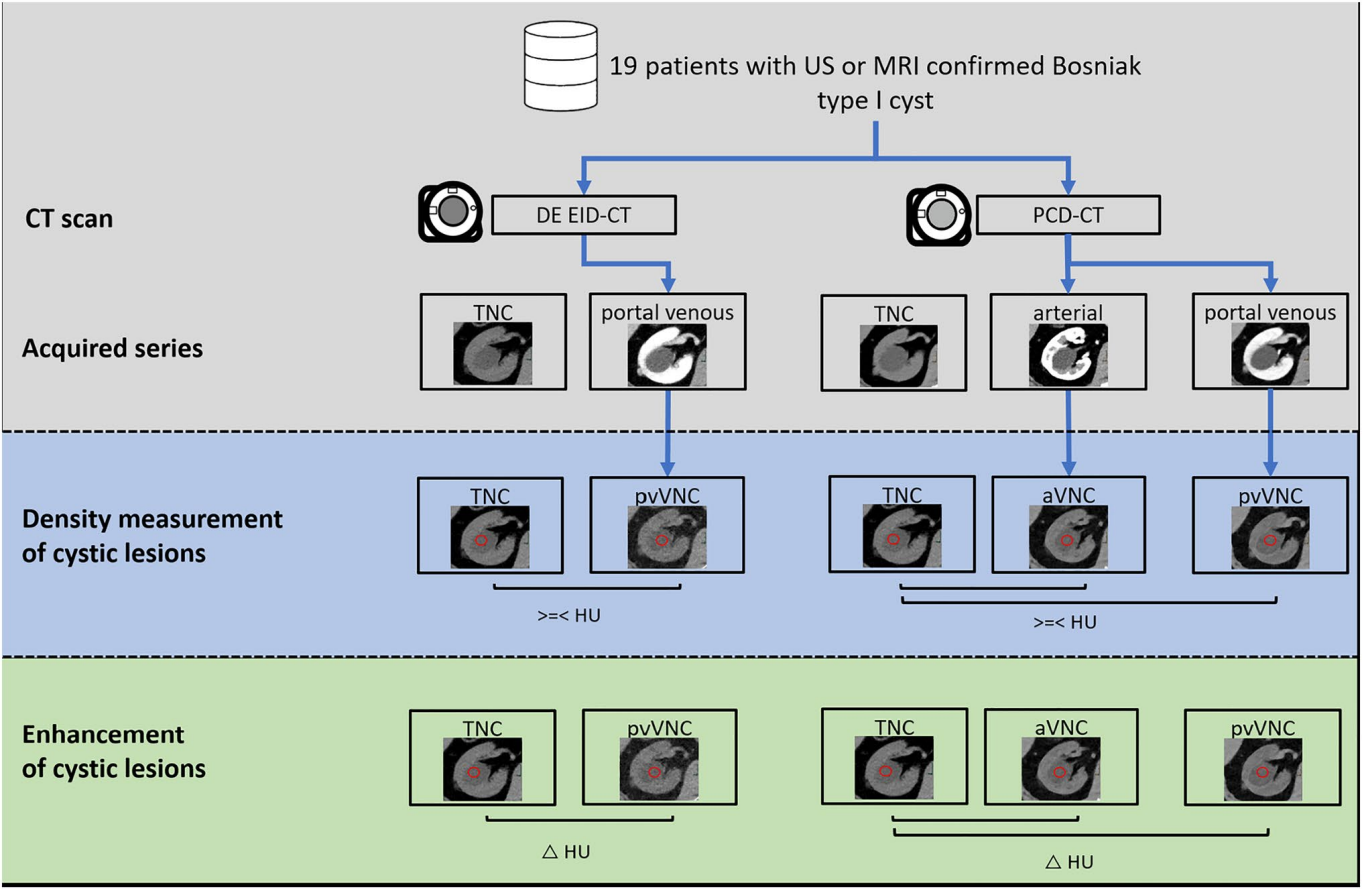
Overview of the study design. All patients received clinically indicated multiphasic abdominal CT in PCD-CT. If a historical multiphasic abdominal CT on DE EID-CT was not already available, the clinically indicated follow-up examination was performed on DE EID-CT. aVNC = arterial virtual non-contrast; pvVNC = portal venous non-contrast; TNC = true non-contrast; PCD-CT = photon-counting detector CT; DE EID-CT = dual-energy integrating detector CT; MRI = Magnetic resonance imaging; US = Ultrasound

Consecutive patients who underwent clinically indicated contrast-enhanced CT of the abdomen were prospectively included in this study between December 2021 and June 2023. Inclusion criteria comprise the presence of a simple renal cystic lesion ≥ 10 mm in diameter and an available multiparametric MRI or ultrasound within reasonable temporal distance of the CT serving as reference standard. Patients were excluded if they had contraindications for contrast-enhanced CT imaging such as allergy to iodine contrast agent, renal impairment, and thyroid dysfunction, as well as individuals under 18 years of age. An overview of the study design is given in Fig. 1.

The local Institutional Review Board (Ethics Committee of the University Medical Center Freiburg, case number 21–2469) approved this prospective study and written informed consent was obtained from all patients before study inclusion.

### CT imaging acquisition and reconstruction

**PCD-CT:** All PCD-CT scans were obtained on a first-generation dual source PCD-CT scanner (NAEOTOM Alpha, Siemens Healthineers, Forchheim, Germany). The imaging protocol comprised a true non-contrast phase, an arterial phase, and a portal venous phase. First, the non-contrast scan was acquired followed by the arterial phase scan, which used contrast agent bolus tracking in the descending aortic for scan initiation after exceeding a threshold of 100 HU. For the portal venous scan, a fixed bolus delay of 80 s after contrast agent administration was used. Contrast agent (Iopromide, Ultravist 370 mg iodine/mL, Bayer Healthcare, Leverkusen, Germany) was administered using a dual-syringe power injector (Accutron CT-D Vision, Medtron, Saarbrücken, Germany) with a body weight adapted amount of contrast agent (1.2 mg/kg) followed by a 40 mL isotonic saline flush with a flow rate of 4.0 mL/s each. The acquisition parameters were as follows: tube voltage 120 kVp, automated attenuation-based tube current modulation with an image quality level (IQ Level) of 100 for non-contrast and arterial phase and 145 for the portal venous phase (99 effective mAs in non-contrast and 100 mAs in arterial and portal venous scans), pitch factor 0.8, collimation 144 × 0.40 mm, rotation time 0.5 s.

From the acquired spectral data of all scans, axial virtual monoenergetic images at 70 keV were calculated using a standard soft tissue kernel (Br40) with a slice thickness of 3.0 mm, an increment of 3 mm and Quantum Iterative Reconstruction (QIR) with strength 4. In addition, VNC images were calculated from the arterial and portal venous images using similar reconstruction parameters.

**DE EID-CT:** All DE EID-CT scans were acquired on a third-generation DE EID-CT scanner (SOMATOM Force, Siemens Healthineers, Forchheim, Germany). The institutional imaging protocol comprised true non-contrast scans, arterial phase scans (using bolus tracking with a threshold of 100 HU and a delay of 15 s) in single-energy mode and a portal venous scans in dual-energy (DE) mode 80 s after body weight adapted (1.2 mg/kg) contrast agent administration (Iopromide, Ultravist 370 mg iodine/mL, Bayer Healthcare, Leverkusen, Germany) using a dual-syringe power injector (Accutron CT-D Vision, Medtron, Saarbrücken, Germany) followed by a saline flush of 40 mL with a flow rate of 4 mL/s similar to the PCD-CT protocol. As the arterial phase scans were acquired in single-energy mode and no VNC reconstructions could be calculated, they were not included in any further analysis.

Acquisition parameters for the true non-contrast images were as follows: 100 kV, automated attenuation-based tube current modulation with a quality reference mAs of 147 mAs, rotation time 0.5 s, pitch factor 0.6, collimation 192 × 0.6 mm. For the portal venous phase reconstruction, the following parameters were used: DE mode with tube voltages of 90 kV and Sn150 kV, automated attenuation-based tube current modulation with quality reference mAs of 152 mAs and 95 mAs, rotation time 0.5 s, pitch factor 0.6, collimation 128 × 0.6 mm.

From the acquired raw data, we reconstructed axial series using a standard soft tissue kernel (Bf40) with 3.0 mm slice thickness and 3 mm increment and Advanced Modeled Iterative Reconstruction (ADMIRE) with strength 3.

In addition, VNC images were calculated from the portal venous series using the same reconstruction parameters.

**Definition and reference imaging for verification of simple cystic lesions:** Cystic renal lesions were defined following the Bosniak classification [7]. A simple renal cystic lesion eligible for inclusion in this study was defined as thin-walled (≤ 2 mm), well-defined, round/oval homogeneous fluid filled lesion with no septation, calcification or solid components and no enhancement (≤ 20 HU on CT).

In all patients, the presence of a simple renal cystic lesion was verified by additional renal imaging (ultrasound or MRI) serving as reference standard.

All ultrasound examinations were performed by board-certified specialists. Simple cystic lesions were defined as oval/round anechoic collections with a thin and smooth wall, no internal flow, no septations or solid components and presence of posterior acoustic enhancement.

MRI was performed as a multiparametric protocol comprising axial and coronal T2-weighted imaging, diffusion-weighted imaging and contrast-enhanced sequences on 1.5 T or 3 T scanners. Simple cystic lesions were diagnosed if the lesion showed high signal on T2-weighted images, no contrast enhancement or diffusion restriction and no septation or solid components. Simple cystic lesions with internal hemorrhage were defined if a lesion presented with high signal on unenhanced T1-weighted imaging, no contrast enhancement and no diffusion restriction. All MRI scans were interpreted by board-certified radiologists in a routine clinical reading session unrelated to the current study.

### Image analysis

If a simple renal cystic lesion > 1 cm was present and verified by MRI or ultrasound, the value of VNC images was measured from arterial phase PCD-CT (aVNC PCD-CT), portal venous PCD-CT (pvVNC PCD-CT) and pv DE EID-CT (pvVNC DE EID-CT) images to compare the absolute density values and the enhancement in a two-step approach.

**Fig. 2:**
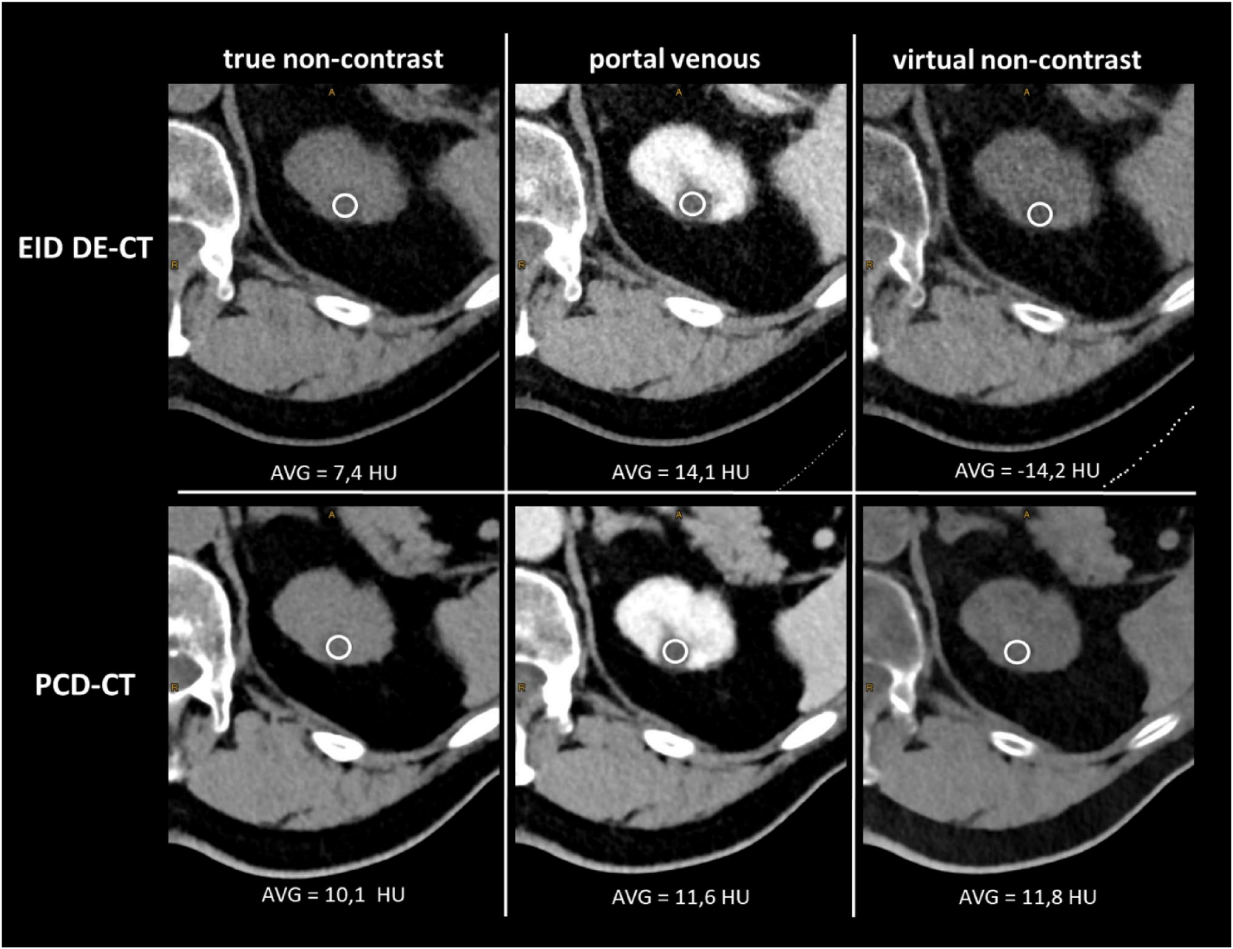
Example of Density-measurements in a simple renal cyst on EID DE-CT and PCD-CT images using true non-contrast, portal venous and virtual non-contrast derived images (from left to right). While true enhancement is < 20 HU, EID DE-CT VNC-derived values are remarkably low leading to false-positive enhancement > 20 HU. PCD-CT = photon-counting detector CT; DE EID-CT = dual-energy integrating detector CT

**Density measurements of cystic lesions:** In a first step, density measurements were performed in true non-contrast (PCD-CT and DE EID-CT) and VNC reconstructions (aVNC PCD-CT, pvVNC PCD-CT, pvVNC DE EID-CT) by placing identical regions of interest (ROIs) with a size of 100 mm^2^ in the same area of the cysts. Subsequently, the mean HU values and standard deviations (SD) were obtained from these measurements (example given in Fig. 2). All measurements were performed by the same radiologists (SR) with 4 years of experience in abdominal CT imaging.

**Enhancement of cystic lesions:** Cystic lesions can still be classified as simple if they have a density ≥ 20 HU on non-contrast scans (most likely due to intralesional hemorrhage) but show no enhancement (defined as 20 HU) on contrast-enhanced scans. To account for this in a second step, the enhancement of the cystic lesions was assessed between the contrast-enhanced series (arterial and portal venous PCD-CT images and portal venous DE EID-CT images) and the true non-contrast images as well as between the contrast-enhanced series and the corresponding VNC reconstructions (aVNC PCD-CT, pvVNC PCD-CT, pvVNC DE EID-CT) using the following equation:

1 $$ {\text{Contrast enhancement}} \left( {\Delta {\text{HU}}} \right) = {\text{contrastenhanced series}} \left( {{\text{HU}}} \right) - {\text{corresponding VNC series}} \left( {HU} \right) $$

### Radiation dose

Radiation doses of the PCD-CT and EID-CT examinations were assessed and compared via the volume CT dose index (CTDI_vol_ [mGy]) in the non-contrast and portal venous series. The investigated protocols were optimized for abdominal/renal indication specific imaging in clinical routine and not with respect to radiation dose.

### Statistical analysis

All statistical analyses were performed using R Foundation for Statistical Computing (Version 4.2.1, Vienna, Austria). Continuous variables are presented as mean ± SD or median and interquartile ranges (IQR) as appropriate. For categorical variables, frequencies and percentages are reported. Density measurements of the PCD-CT series (true non-contrast, aVNC, pvVNC) were compared via repeated measure ANOVA and post-hoc pairwise comparison using the Tukey method. For DE EID-CT density measurements (true non-contrast vs. pvVNC), paired sample *t* tests were conducted. To evaluate a potential cystic enhancement, the absolute difference between the true non-contrast and corresponding VNC reconstruction was calculated.* p* values were considered statistically significant if < 0.05.

## Results

### Patients characteristics

**Fig. 3:**
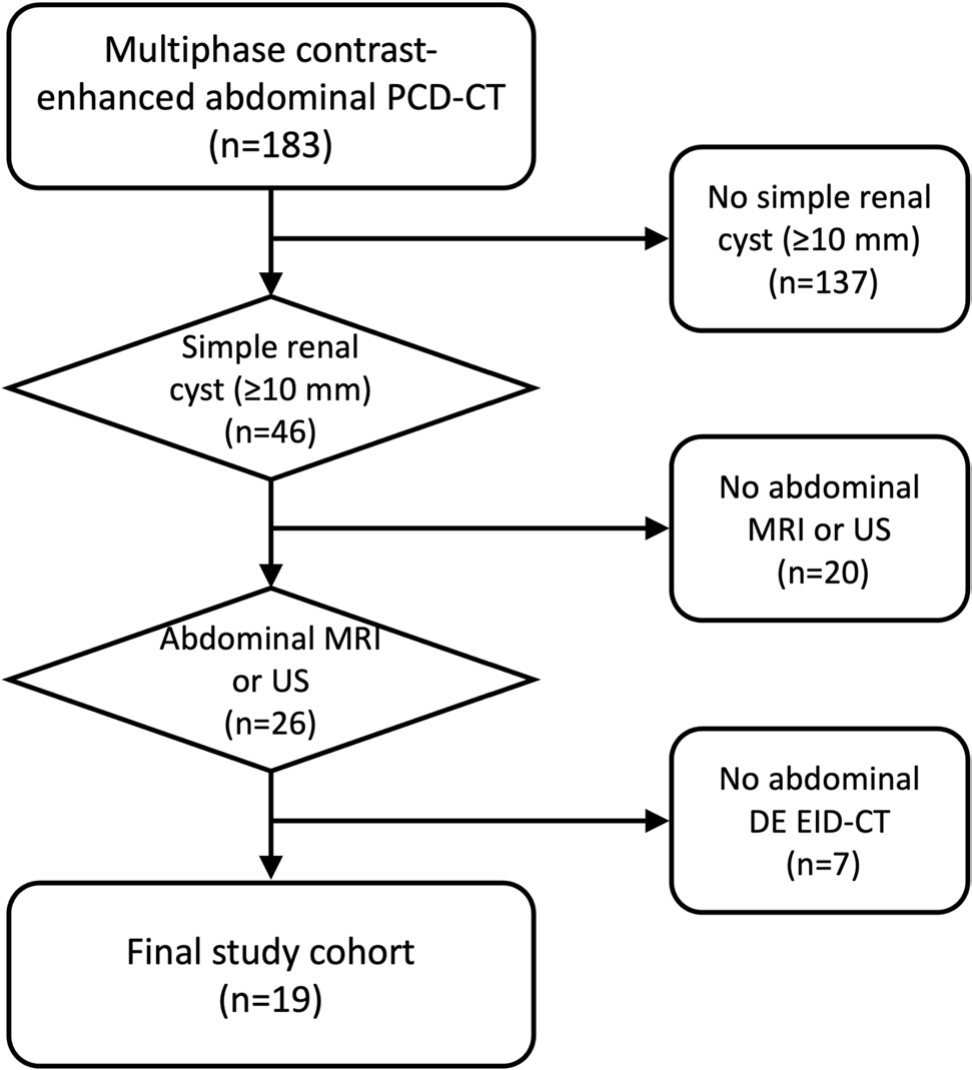
Consort diagram. PCD-CT = Photon-counting detector CT; DE EID-CD = Dual-energy integrating detector CT; MRI = Magnetic resonance imaging; US = Ultrasound

Between 02/2022 and 05/2023, 183 patients with oncologic diseases who underwent clinically indicated multiphase contrast-enhanced PCD-CT were prospectively included in this study. 137 patients had to be excluded because no simple cystic renal lesion ≥ 10 mm in diameter was present. For an additional 15 patients, no MRI was available and another 5 had no abdominal ultrasound examination. Finally, for 7 patients, no DE EID-CT was available resulting in a final study cohort of 19 patients (see Fig. 3).

The mean age 69.5 ± 10.7 years; 89.5% (*n* = 17) were male and the mean BMI was 26.50 ± 3.7 kg/m^2^. The presence of a simple cystic renal lesion was verified by multiparametric MRI in *n* = 15 patients and in *n* = 4 via ultrasound within 74.0 ± 59.7 weeks of CT imaging. Further detailed patient characteristics are presented in Table 1.

**Table 1:** Patient and imaging characteristics

*Characteristics*	
Age (years)	69.5 ± 10.7
Sex	17 male (89.5%)
BMI (kg/m2)	26.5 ± 3.7
Time interval between PCD-CT and DE EID-CT (weeks)	39.9 ± 30.1
Simple renal cystic lesion size (mm)	22.3 ± 9.9
*PCD-CT scans*	
True non-contrast CTDIvol (mGy)	9.53 ± 3.65
Arterial CTDIvol (mGy)	7.80 ± 3.13
portal venous CTDIvol (mGy)	8.66 ± 2.31
*DE EID-CT scans*	
True non-contrast CTDIvol (mGy)	10.45 ± 3.67
Portal venous CTDIvol (mGy)	10.95 ± 3.27

*PCD-CT* Photon-counting detector CT; *DE EID-CD* Dual-energy integrating detector CT; *BMI* Body mass index; *CTDIvol* volume CT dose index

### Image analysis

**Density measurements of cystic lesions:** Density measurements in the true non-contrast, aVNC and pvVNC PCD-CT reconstructions were 13.7 ± 11.6 HU, 9.27 ± 10.1 HU and 12.3 ± 9.9 HU, respectively. For DE EID-CT, mean HU values for true non-contrast were 12.8 ± 9.7 and 6.3 ± 14.9 for pvVNC images.

One-way ANOVA revealed no significant effect for the types of reconstruction on HU values in PCD-CT (F(2, 34.7) = 1.24, *p* = 0.301). In contrast, a significant difference between true non-contrast vs. pvVNC images was found for DE EID-CT (*p* = 0.02).

**Enhancement of cystic lesions:** To investigate whether VNC reconstructions allow for assessing a potential contrast enhancement of lesions, delta HU values between the contrast-enhanced and VNC reconstructions for PCD-CT and DE EID-CT were calculated and compared to delta HU values using true non-contrast reconstructions serving as reference.

For PCD-CT, enhancement for both aVNC and pvVNC reconstructions was < 20 HU for all evaluated cystic lesions (aVNC 4.5 ± 5.0 HU [range − 6.2 to 14.2] and pvVNC 2.4 ± 5.3 HU [range − 6.4 t 13.5]), which was similar to using true non-contrast images (true non-contrast for arterial 0.0 ± 1.97 [range − 2.7 to 4.7] HU and true non-contrast for portal venous 0.7 ± 2.4 HU [range − 4.7 to 3.6]).

**Fig. 4:**
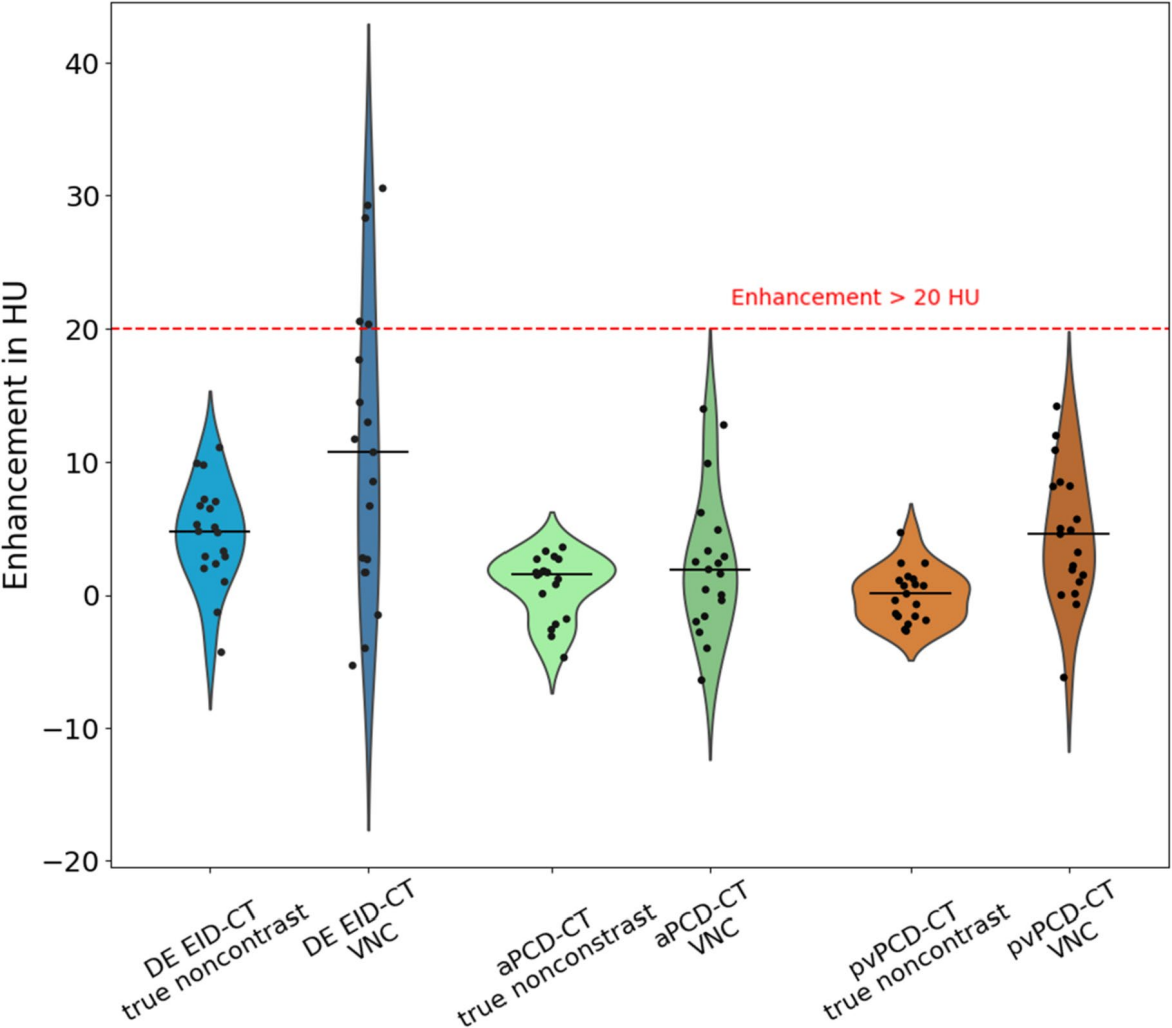
Results of enhancement analysis between true non-contrast and VNC reconstructions for DE EID-CT and PCD-CT. Whereas enhancement for both aVNC and pvVNC reconstructions was < 20 HU for all evaluated cystic lesions using PCD-CT, which was confirmed by true non-contrast images (green and brown violin plots), enhancement for DE EID-CT VNC reconstructions was > 20 HU in five cases, which was not seen for the true non-contrast DE EID-CT images, indicating that these lesions would have been erroneously classified as complicated using the DE EID-CT VNC approach. VNC = virtual non-contrast; aVNC = arterial virtual non-contrast; pvVNC portal venous non-contrast; PCD-CT = photon-counting detector CT; DE EID-CT = dual-energy integrating detector CT

DE EID-CT measurements revealed an enhancement of > 20 HU in five lesions (26.3%) using the pvVNC reconstructions (pvVNC 11.0 ± 11.1 HU [range − 5.3 to 30.5]), which was not seen with the true non-contrast images (4.6 ± 3.8 HU [range − 4.3 to 11.1]) (see Fig. 4).

### Radiation dose

Radiation dose analysis revealed no significant differences of the CTDI_vol_ for non-contrast scans 9.53 vs. 10.45 mGy (*p* = 0.208), whereas a small but significant difference with a lower radiation dose for PCD-CT vs. DE EID-CT was noted for portal venous phase series (8.66 vs. 10.95 mGy; *p* < 0.001).

## Discussion

In this study, we investigated the potential of PCD-CT VNC reconstructions for characterization of simple cystic renal lesions in comparison with conventional DE EID-CT VNC reconstructions. PCD-CT-derived VNC images allowed for a more accurate measurement of lesion density and contrast enhancement resulting in a correct characterization of all analyzed lesions whereas DE EID-CT VNC images erroneously classified 26.3% of lesions as complicated.

This is of clinical importance as cystic renal lesions are frequent incidental findings and thus accurate characterization is crucial for patient management [9]. While simple cystic lesions require no further workup, a density > 20 HU on non-contrast scans or contrast enhancement > 20 HU indicates potentially solid lesions, which need dedicated investigation to rule out malignancy [6, 7]. As of now, acquisition of a multiphase protocol comprising true non-contrast and contrast-enhanced series is recommended for dedicated renal imaging to accurately assess lesion density and lesion enhancement [8, 16]. With the introduction of DE-CT systems and the feasibility of calculating VNC images, a new approach for characterizing incidentally detected lesions on contrast-enhanced scans has become available [10]. While initial expectations were high that non-contrast scans could be omitted, a growing body of research indicated that DE EID-CT VNC images are of limited value given substantial differences in HU measurements compared to true non-contrast images. For example, a previous study by Meyer et al. reported a difference in HU values between true and virtual non-contrast images of 7.4 HU ± 7.2 in unenhanced, low-attenuation lesions and 11.6 HU ± 10.2 HU in unenhanced, high-attenuation lesions with a maximum difference in attenuation between VNC and true non-contrast images of up to 48.1 HU [13]. Another analysis by Cao et al. of 86 cysts found false-positive enhancement in DE-CT-derived VNC greater than 20 HU in 9% of simple and in 27% of hyperattenuating cysts [12]. Erroneous classification has direct implication on patients care, as a false-positive apparent enhancement would characterize a lesion as potentially malignant resulting in unnecessary workup and costs [9]. Our results are in line with these findings as DE EID-CT VNC images revealed false-positive enhancement in 26%.

In contrast, in the same patient, population PCD-CT-derived VNC images allowed for accurate characterization of all included renal lesions as simple cystic lesions likely due to the perfect spatial and temporal registration of the spectral information, as well as further improved properties of the acquired signal, like the absence of electronic noise. This novel detector technology thus offers the promising possibility to overcome the limitations of DE EID-CT VNC reconstructions with the potential to substitute true non-contrast series by VNC reconstructions without compromising diagnostic accuracy. Our results are supported by a pre-clinical study from Boll et al. who reported that spectral CT has the potential to enable distinct characterization of hyperattenuating fluids in a renal phantom through accurate assessment of their levels of attenuation [17]. Furthermore, high reliability of VNC reconstruction in clinical PCD-CT was also demonstrated in a recently published study on 100 patients, in which accurate parenchymal attenuation-values from VNC images compared to true non-contrast images in arterial or portal venous phase CT was noted [18].

The intrinsic spectral data acquired in every PCD-CT scan allows for calculating VNC whenever necessary [14]. In contrast, on energy integrating (EID)-CT systems, the DE mode needs to be selected prior to the examination. Moreover, PCD-CT had a significantly lower radiation dose compared with EID-CT in our study [15].

The PCD-CT will have great impact on clinical routine, though the full potential is not yet exploited, and PCD-CT could yield further technical advances. In animal studies, spectral discrimination was successfully used to virtually subtract multiple contrast agents simultaneously. Thus, for example, VNC, arterial and portal venous contrast could be acquired within one scan, providing a one-stop-shop solution for many examinations [19].

Besides the fact that our results need to be confirmed in a larger cohort, our study has the following limitations. The diagnostic accuracy of PCD-CT VNC reconstructions needs to be determined not only for characterization of simple cystic lesions but also for complex cysts and malignant renal lesions with special regards to false-negative results. In addition, we only analyzed lesions with a diameter > 10 mm. Whether VNC reconstructions facilitate reliable HU measurements in smaller lesions remains to be investigated. Finally, acquisition and reconstructions parameters slighted differed between the PCD-CT and EID-CT imaging protocol due to differences in scanner software and reconstruction techniques and no dual-energy data were acquired the arterial scan in the DE EID-CT.

In conclusion, PCD-CT-derived VNC images allow for reliable and accurate characterization of simple cystic renal lesions similar to true non-contrast scans whereas VNC images calculated from DE EID-CT resulted in false characterization in 26%. Thus, PCD-CT-derived VNC images may serve as a decision support tool in clinical routine with the potential to substitute for true non-contrast images and reduce radiation dose and follow-up imaging.

## Abbreviations

VNC: Virtual non-contrast
aVNC: Arterial virtual non-contrast
pvVNC: Portal venous non-contrast
PCD-CT: Photon-counting CT
DE EID-CT: Dual-energy integrating detector CT
DE: Dual energy
QIR: Quantum iterative reconstruction
ROI: Region-of-interest
CTDIvol: Volume CT dose index
